# A study on the knowledge and attitudes on advanced life support among medical students and medical officers in a tertiary care hospital in Sri Lanka

**DOI:** 10.1186/s13104-016-2270-5

**Published:** 2016-10-12

**Authors:** Dissanayake Mudiyanselage Priyantha Udaya Kumara Ralapanawa, Kushalee Poornima Jayawickreme, Ekanayake Mudiyanselage Madhushanka Ekanayake, Pallegoda Vithanage Ranjith Kumarasiri

**Affiliations:** 1 Department of Medicine, University of Peradeniya, Peradeniya, Sri Lanka; 2 Department of Community Medicine, University of Peradeniya, Peradeniya, Sri Lanka

**Keywords:** Advanced life support, Cardiopulmonary resuscitation, Knowledge, Attitudes, Skills, Medical education

## Abstract

**Background:**

Advanced life support (ALS) and cardio pulmonary resuscitation, provided at the right time is essential for improving mortality in medical emergencies. Accurate knowledge and skills on this regard, in all medical personals is an essential part of medical education and it should be up to date with varying protocols. The aim of this study is to assess the knowledge and attitudes among the undergraduate medical students and medical officers in the Teaching Hospital Peradeniya and provide suggestions to improve the training programme on ALS.

**Methods:**

A standardized self-administered questionnaire regarding knowledge and attitudes on ALS was filled by 4th and final year medical students, and medical officers, and the data was analyzed.

**Results:**

There were 411 eligible candidates and of them 130 (31.6 %) were 4th year medical students, 221 (53.8 %) were final year medical students and 60 (14.6 %) were medical officers. Of the medical officers, only 15.8 % indicated that the internship training was adequate to handle an emergency confidently. Approximately 45 % of the medical officers and 34.6 % of the final year medical students were confident of saving lives with their current ALS knowledge. However, only 22 % of 4th year medical students were confident in saving the life of a patient.

**Conclusions:**

Overall, just over 10 % of participants demonstrated inadequate ALS knowledge scores. A significantly higher proportion of final year medical students had good knowledge, compared to medical officers and 4th year students. Only one-third of participants were confident in saving a life with their current ALS knowledge. Nearly all participants thought that the ALS course should be reevaluated frequently.

## Background

Advanced life support (ALS) and cardio pulmonary resuscitation (CPR), provided at the right time greatly help in improving mortality in medical emergencies. Accurate knowledge regarding ALS for all medical personals is an essential part of medical education and it should be up to date with varying protocols according to evidence [1, 2]. The lack of training and inability to cope with medical emergencies can lead to tragic consequences and probable legal hazards [3]. Studies conducted in the past show a lack and inconsistency in knowledge regarding the CPR and defibrillation [4]. Therefore, it is of paramount importance to improve awareness among all medical practitioners, especially among those outside the tertiary healthcare facilities. By this means, medical professionals must be well prepared, competent and confident to deal with medical emergencies [3]. Medical students are educated and trained on ALS at the commencement of the 4th year, and all pre-intern doctors undergo an ALS training program organized by the Sri Lanka Medical Council prior to the commencement of their medical internship. Good knowledge and attitudes in medical professionals contributes to the successful outcome of ALS globally and thereby reducing mortality following medical emergencies. The main purpose of this study is to assess the knowledge and attitudes among the undergraduate medical students and medical officers in the Teaching Hospital, Peradeniya, Sri Lanka and give suggestions to improve the undergraduate and postgraduate training programme on ALS. In addition to that, we expect to emphasize the value of refreshing knowledge on ALS among the medical officers with rapidly changing protocols of the ALS guidelines.

## Methods

### Study setting

The current study was a cross sectional survey carried out among 421 subjects to assess their knowledge and attitudes on ALS. The study sample comprised medical students of the 4th and final years and pre-intern doctors at the Faculty of Medicine, University of Peradeniya, and medical officers ranging from intern house officers, Senior House Officers, Registrars to Senior Registrars of the Teaching Hospital Peradeniya. Consultants were not included in this study sample.

### Data collection

Data collection commenced after obtaining the approval from the institutional Ethical Review Committee of the Faculty of Medicine, University of Peradeniya.

Data collection was done by a self-administered standardized questionnaire (Appendix 1) prepared based on the United Kingdom resuscitation council guidelines on ALS [5]. The questionnaire comprised 10 questions regarding knowledge and seven questions regarding attitudes. Data was collected from selected participants, who gave informed written consent. Incomplete response sheets were excluded from data capturing the analysis.

### Statistical analysis

The basis of data analysis was to assess retention of knowledge and skills, rate of decay over time and relationship between clinical exposure and decay. Data was entered in a password protected computer on Microsoft Excel and it was analyzed using a standard analyzing technique; SPSS 20 (Statistical Package for Social Sciences 20.0). Monovariable analysis was done using tables and charts and compared according to the percentage and central tendencies. Bivariable analysis was done to see the association by using two by two table and significance was analyzed by using chi-square significant test.

## Results

### Basic demography

The total number of potential study population was 497. Total number of respondents was 421 (84.7 %). Ten (10) questionnaires which were incomplete were rejected and not included in the data analysis. There were 411 eligible candidates and of them 130 (31.6 %) were 4th year medical students, 221 (53.8 %) were final year medical students and 60 (14.6 %) were medical officers working at the Teaching Hospital Peradeniya. The total sample included 192 (46.7 %) of males and 219 (53.3 %) of females. The age of the subjects ranged from 21 to 57 years (mean 27.55 ± 2.44 years). Majority (65 %) of doctors were in the service more than 10 years (Table 1). The knowledge score was inadequate (<50 %) among 12.7 % of the sample. Approximately 43.1 % had good knowledge on ALS. The mean score was 67.61 % with a and SD of 16.45 (n = 411) (Table 2).

**Table 1:** Demographic distribution of study population

Variables	Categories	Number (%)
Age	<24 years	130 (31.6 %)
	24–29 years	226 (55.0 %)
	>30 years	55 (13.4 %)
Gender	Male	192 (46.7 %)
	Female	219 (53.3 %)
Position	4th year medical students	130 (31.6 %)
	Final year medical students	221 (53.8 %)
	Medical officers	60 (14.6 %)
Duration of work (medical officers)	<10 years	21 (35.0 %)
	>10 years	39 (65.0 %)

**Table 2:** Knowledge score distribution

Knowledge score %	Frequency	Percentages (%)
Poor (<50)	52	12.7
Average (50–69)	182	44.3
Good (≥70)	177	43.1

Of the medical officers, only 15.8 % indicated that the internship training was adequate to handle an emergency confidently. A majority of 98 % supported the importance of having ALS training before practicing as a medical officer. Importance of teaching ALS during undergraduate training was identified by 96 % of the subjects. Strangely 55.5 % of the participants mentioned that they were not confident enough in selecting or giving emergency drugs. A majority of 94.5 % of participants thought that the ALS course should be reevaluated frequently. Only 32.1 % of participants were confident in saving a life with their current ALS knowledge (Table 3).

**Table 3:** Frequency distribution of attitudes toward ALS

Attitude questions	Answers
I feel that my internship training is adequate to equip me to handle resuscitation confidently (n = 57)	Yes 9 (15.8 %)
	No 43 (75.4 %)
	Not sure 5 (8.8 %)
Resuscitation should be initiated by a senior Medical Officer (n = 393)	Yes 61 (15.5 %)
	No 308 (78.4 %)
	Not sure 24 (6.1 %)
All junior doctors should have Advanced Life Support (ALS) course training before practice (n = 399)	Yes 391 (98.0 %)
	No 4 (1.0 %)
	Not sure 4 (1.0 %)
ALS course should be taught during undergraduate years (n = 400)	Yes 385 (96.3 %)
	No 10 (2.5 %)
	Not sure 5 (1.3 %)
Are you confident enough in selecting or giving emergency drugs (n = 402)	Yes 84 (20.9 %)
	No 223 (55.5 %)
	Not sure 95 (23.6 %)
Do you think that the ALS course should be reevaluted frequently (n = 397)	Yes 375 (94.5 %)
	No 22 (5.5 %)
Do you think that you are confident enough in saving a life with your ALS knowledge (n = 399)	Yes 128 (32.1 %)
	No 130 (32.6 %)
	Not sure 141 (35.3 %)

Only 25.5 % of above 30 years of age category of participants had good knowledge on ALS. Corresponding figures for 24–29 and <24 were 47.8 and 42.3 % respectively. These differences were statistically significant. A slightly higher proportion of males had good knowledge on ALS than females; however the difference was not statistically significant. Significantly higher proportion (56 %) of final year medical students had good ALS knowledge, whereas 27 and 29 % of medical officers and the 4th year medical students had good ALS knowledge respectively. 42.8 % of medical officers with less than 10 years of experience had good ALS knowledge. However, 20.5 % of medical officers with more than 10 years of service had good knowledge, although work in specialities with frequent exposure to ALS activities, frequent exposure to ALS activities did not improve the knowledge of ALS among medical officers (Table 4). Among the medical officers working in the specialities where ALS was frequently encountered and 29 % of them had good knowledge. This proportion was 25 % for the medical officers not working in specialities where ALS was frequently encountered. This difference was not statistically significant.

**Table 4:** Distribution of knowledge scores by demographic variables and duration of work experience

	Knowledge score categories			*P* value*
	Poor (<50)	Average (50–69)	Good (≥70)	
Age (411)				
<24 years (n = 130)	17 (13.1 %)	58 (44.6 %)	55 (42.3 %)	<0.001
24–29 years (n = 226)	15 (6.6 %)	103 (45.6 %)	108 (47.8 %)	
≥30 years (n = 55)	20 (36.4 %)	21 (38.2 %)	14 (25.5 %)	
Sex (n = 411)				
Male (n = 192)	3 (12.0 %)	83 (43.2 %)	86 (44.8 %)	0.791
Female (n = 219)	29 (13.2 %)	99 (45.2 %)	91 (41.6 %)	
Position (n = 411)				
4th years (n = 130)	22 (16.9 %)	71 (54.6 %)	37 (28.5 %)	<0.001
Final years (n = 221)	9 (4.1 %)	88 (39.8 %)	124 (56.1 %)	
Medical officers (n = 60)	21 (35.0 %)	23 (38.3 %)	16 (26.7 %)	
Duration of work as a medical officer (n = 60)				
<10 years (n = 21)	7 (33.4 %)	5 (23.8 %)	9 (42.8 %)	0.139
>10 years (n = 39)	14 (35.9 %)	17 (43.6 %)	8 (20.5 %)	
Specialty (n = 59)				
Specialties where ALS frequently encountered (n = 31)	12 (38.7 %)	10 (32.3 %)	9 (29.0 %)	0.524
Specialties where ALS frequently not encountered (n = 28)	8 (28.6 %)	13 (46.4 %)	7 (25.0 %)	

* P value was calculated by using Chi square test

Approximately 77 % of ≥30 years aged participants and 50 % of 20–29 years of age participants felt that the internship training on ALS was inadequate. Significantly high (50.9 %) proportion of ≥30 years of participants were confident of selecting and giving emergency drugs. The results showed that only 11.3 and 19.1 % of <24 and 24–29 years of age participants were confident of selecting and giving emergency drugs. Over 90 % of participants in each of the age categories thought that frequent re evaluation of ALS course was necessary. Approximately 47 % of the participants ≥30 years of age indicated that they were confident of saving lives with their current ALS knowledge (Table 5).

**Table 5:** Distribution of attitudes towards ALS by age categories of participants

	Age categories			*P* value*
	<24 years	24–29 years	≥30 years	
1). I feel that my internship training is adequate to equip me to handle resuscitation confidently (n = 57)				
Yes		1 (25.0 %)	8 (15.1 %)	0.388
No		2 (50.0 %)	41 (77.4 %)	
Not sure		1 (25.0 %)	4 (7.5 %)	
Total		4 (7.01 %)	53 (92.99 %)	
5). Are you confident enough in selecting or giving emergency drugs (n = 402)				
Yes	14 (11.3 %)	43 (19.1 %)	27 (50.9 %)	<0.001
No	84 (67.7 %)	119 (52.9 %)	20 (37.7 %)	
Not sure	26 (21.0 %)	63 (28.0 %)	6 (11.3 %)	
Total	124 (30.8 %)	225 (56.0 %)	53 (13.2 %)	
6). Do you think that the ALS course should be re-evaluated frequently (n = 397)				
Yes	113 (93.4 %)	210 (94.2 %)	52 (98.1 %)	0.438
No	8 (6.6 %)	13 (5.8 %)	1 (1.9 %)	
Total	121 (30.5 %)	223 (56.2 %)	53 (13.4 %)	
7). Do you think that you are confident enough in saving a life with your ALS knowledge (n = 399)				
Yes	28 (22.8 %)	75 (33.6 %)	25 (47.2 %)	0.023
No	47 (38.2 %)	68 (30.5 %)	15 (28.3 %)	
Not sure	48 (39.0 %)	80 (35.9 %)	13 (24.5 %)	
Total	123 (30.8 %)	223 (55.9 %)	53 (13.3 %)	

* P value was calculated by using Chi square test

A significantly higher proportion of males (28.3 %) were confident to selecting and giving emergency drugs than female (14.4 %) participants. The sex of the participant did not significantly differ when the internship training on ALS and re-evaluation of the ALS courseware concerned. Significantly high proportion of male participants (41.6 %) thought that they were confident in saving a life with their current ALS knowledge. The corresponding figure for female participants was 23.8 % (Table 6).

**Table 6:** Sex distribution of attitudes towards ALS

	Sex		*P* value*
	Male	Female	
1). I feel that my internship training is adequate to equip me to handle resuscitation confidently (n = 57)			
Yes	5 (20.0 %)	4 (12.5 %)	0.141
No	16 (64.0 %)	27 (84.4 %)	
Not sure	4 (16.0 %)	1 (3.1 %)	
Total	25 (100)	32 (100)	
5). Are you confident enough in selecting or giving emergency drugs (n = 402)			
Yes	53 (28.3 %)	31 (14.4 %)	<0.001
No	106 (56.7 %)	117 (54.4 %)	
Not sure	28 (15.0 %)	67 (31.2 %)	
Total	187 (100)	215 (100)	
6). Do you think that the ALS course should be re-evaluated frequently (n = 397)			
Yes	175 (94.6 %)	200 (94.3 %)	Fishers exact test (2 tailed) 1.000
No	10 (5.4 %)	12 (5.7 %)	
Total	185 (100)	212 (100)	
7). Do you think that you are confident enough in saving a life with your ALS knowledge (n = 399)			
Yes	77 (41.6 %)	51 (23.8 %)	<0.001
No	56 (30.3 %)	74 (34.6 %)	
Not sure	52 (28.1 %)	89 (41.6 %)	
Total	185 (100)	214 (100)	

* P value was calculated by using Chi square test

Approximately 46.6, 21.4 and 8 % of medical officers, final year medical students and 4th year medical students indicated that they were confident of selecting and giving emergency drugs. All categories of participants agreed for the re-evaluation of the ALS courses frequently. Approximately 45 % of the medical officers and 34.6 % of the final year medical students were confident of saving lives with their current ALS knowledge. However, only 22 % of 4th year medical students were confident in saving the life of a patient (Table 7).

**Table 7:** Distribution of attitudes towards ALS by work experience of participants

	Position			*P* value*
	4th years	Final years	Medical officers	
5). Are you confident enough in selecting or giving emergency drugs (n = 402)				
Yes	10 (8.0 %)	47 (21.4 %)	27 (46.6 %)	<0.001
No	85 (68 %)	114 (52.1 %)	24 (41.4 %)	
Not sure	24 (24.0 %)	58 (26.5 %)	7 (12.1 %)	
Total	125 (100.0)	219 (100)	58 (100)	
6). Do you think that the ALS course should be re-evaluated frequently (n = 397)				
Yes	109 (90.1 %)	209 (95.9 %)	57 (98.3 %)	0.032
No	12 (9.9 %)	9 (4.1 %)	1 (1.7 %)	
Total	121 (100)	218 (100)	58 (100)	
7). Do you think that you are confident enough in saving a life with your ALS knowledge (n = 399)				
Yes	27 (21.8 %)	75 (34.6 %)	26 (44.8 %)	0.016
No	48 (38.7 %)	64 (29.5 %)	18 (31.0 %)	
Not sure	49 (39.5 %)	78 (35.9 %)	14 (24.1 %)	
Total	124 (100)	17 (100)	58 (100)	

* P value was calculated by using Chi square test

Irrespective of the speciality, whether ALS frequently encountered or not, the medical officers did not show significant difference when selecting and giving emergency drugs, frequent re-evaluation of ALS courses and their confidence in saving a life with their current ALS knowledge (Table 8).

**Table 8:** Distribution attitudes towards ALS by specialty

	Speciality		*P* value*
	Specialties where ALS frequently encountered (31)	Specialties where ALS frequently not encountered (28)	
5). Are you confident enough in selecting or giving emergency drugs (n = 402)			
Yes	13 (44.8 %)	14 (50.0 %)	0.509
No	11 (37.9 %)	12 (42.9 %)	
Not sure	5 (17.2 %)	2 (7.1 %)	
Total	29 (100)	28 (100)	
6). Do you think that the ALS course should be re-evaluated frequently (n = 397)			
Yes	29 (100.0 %)	27 (96.4 %)	Fishers exact test (2 tailed) 0.491
No	0	1 (3.6 %)	
Total	29 (100)	282 (100)	
7). Do you think that you are confident enough in saving a life with your ALS knowledge (n = 399)			
Yes	13 (44.8 %)	12 (42.9 %)	0.767
No	10 (34.5 %)	8 (28.6 %)	
Not sure	6 (20.7 %)	8 (28.6 %)	
Total	29 (100)	28 (100)	

* P value was calculated by using Chi square test

## Discussion

Over a million deaths occur worldwide annually as a result of preventable and reversible critical illness preceded by circulatory shock and respiratory failure [6]. Basic life support (BLS) is the foundation of life saving following out-of-hospital cardiac arrest [7]. Therefore, it is important that even a lay person be competent in applying BLS, while it is important for all medical professionals to be knowledgeable and competent in applying ALS for medical emergencies [3]. The chance of successful resuscitation after sudden cardiac arrest decreased by 7–10 % with every additional minute [8]. The chain of survival in sudden cardiac arrest includes early activation of emergency medical services, early CPR, early defibrillation, and early ALS to reduce death and post-resuscitation care [8].

The guidelines used in Sri Lanka in teaching and training medical students and doctors are guidelines and protocols of the Resuscitation Council of the United Kingdom. Similarly the most recent American Heart Association and European Resuscitation Council ALS guidelines are based on the 2010 International Liaison Committee on Resuscitation (ILCOR) consensus on science and treatment recommendations [9–11]. It is important to be up to date on recent revisions of guidelines.

This study population showed a mean knowledge score of 67.61 and 12.7 % of the population had poor knowledge scores. In contrast a study by Avabratha et al. including medical interns of three medical colleges in coastal Karnataka reported 45.2 % to have poor knowledge regarding resuscitation [12]. On detailed analysis of knowledge scores, there was no statistically significant difference of knowledge scores among males and females. There was a statistically significant difference (<0.001) between knowledge scores among 4th year medical students, final year medical students and medical officers. 56.1 % of final years had good knowledge scores. Almesned et al. also revealed that final year medical students had a better knowledge on BLS than junior medical students [13]. High knowledge scores among final year students can be attributed to them having thoroughly studied and being up to date with the ALS Guidelines. In contrast Akritia et al. reported inadequate knowledge on ALS in undergraduate medical students [14]. 0.42.8 % of medical officers with less than 10 year’s work exposure and 20.5 % of those with more than 10 year’s work exposure respectively had good knowledge scores, though there was no statistically significant association between duration of work and knowledge on ALS. An audit on knowledge on CPR among Intern House Officers at the National Hospital Sri Lanka showed average knowledge scores of 51 % and 55.6 % among interns with work exposure of >6 months and <6 months respectively [15]. Majority of studies reported a decline in knowledge on ALS over time, whilst two studies have shown no decline in knowledge retention during their study period [16, 17]. Hammond et al. reported equivalent knowledge on testing at 18 months, which was attributed to the Hawthorne effect [16]. The patterns and rate of decay of knowledge and skills defers among studies, but the it is most significant within the first 6–12 months following training [18]. 29 % of medical officers of specialties wherein ALS is frequently encountered inclusive of Medicine, Paediatrics, Preliminary care, Anesthesia, and Intensive care units had good knowledge on ALS, whilst 25 % of those of other specialties had good knowledge which showed no statistically significant difference. The previously mentioned audit on knowledge on CPR among Intern House Officers at the National Hospital Sri Lanka showed mean knowledge scores of 58.4, 50 and 43.4 % among specialties of medicine, surgery, obstetrics and gynecology respectively [15]. It is important that medical officers of all specialties are thorough in ALS as cardiac arrest can occur in any field. Therefore, frequent revision of knowledge and continuous practice is important in retaining knowledge and skills on ALS, and updating one’s knowledge on any changes done in guidelines, irrespective of the field of practice.

In addition to having a good knowledge on ALS, it is important to have positive attitudes and confidence in handling such emergency situations. Medical students are taught and trained in ALS during their undergraduate period and was agreed to be important by 96.3 % of the study population. An ALS training programme is carried out annually by the Sri Lanka Medical Council prior to internship, and 98 % agreed on the importance of having such a training programme before commencing practice. A significant number declared not being confident enough to save lives with their current ALS knowledge (32.1 %), and to select or give emergency drugs (55.5 %), which is a drawback in medical practice in handling emergency situations. Narayan et al. reported that 59.9 % had positive attitudes towards BLS, and Roshana et al. showed similar results [3, 19]. Males were reported to be significantly more confident than females in practicing ALS with positive attitudes, though there was no significant difference among the two genders in their knowledge scores, indicating the importance of females to develop more self-confidence and improving their practical skills.

Medical officers of higher age categories, and those with increased years of work experience had better attitudes and confidence in practicing ALS skills in an emergency. A higher proportion of medical officers compared to final year and 4th year students had confidence in selecting and using emergency drugs and saving lives by ALS, with 4th year students had the least confidence level, which can be attributed to the lack of exposure and experience. In contrast to these findings, medical students compared to medical officers had better knowledge on ALS, with their knowledge level decreasing with the number of years of work experience. This shows that the knowledge is the highest at the time of training, followed by decline of knowledge due to the absence of frequent revisions. A majority of 75.4 % of medical officers stated that the internship training is not adequate to handle resuscitation confidently, with majority sharing that thought being females, and those with a longer work experience, implying that teaching and training should be commenced during the undergraduate years and continued at repeated intervals after internship.

Sudeep et al. demonstrated that training improved the knowledge and skills of CPR [20]. In addition, Jensen et al. showed that at least half a year of clinical experience was important before participating in an ALS course in order to improve the retention of learning [21]. A previous study has shown that self-directed learning lead to improved 6 months of retention of knowledge [22]. Worldwide resuscitation councils recommend that healthcare providers should receive ALS retraining or refresher courses every 2 years or longer [18]. Kromann et al. demonstrated the effectiveness of the “testing effect”, which showed that testing as the final step of a resuscitation course increased learning outcomes [23, 24]. The American Heart Association suggests that skills training requires more frequent assessment, preferably within the 2 year certification, with reinforcement as necessary [18]. A significant proportion of 94.5 % of this study population, irrespective of age, sex or specialty agreed that the ALS course should be re-evaluated frequently, which needs to be implemented in our practice. A study done in 2013 on the effectiveness of “Train the Trainer” model of resuscitation education for rural peripheral hospital doctors in Sri Lanka, revealed the deficit in resuscitation training in the rural Sri Lankan setting, and showed that this model was effective in improving resuscitation knowledge and skills among doctors in peripheral hospitals [25].

## Limitations

Practical skills of ALS could not be assessed in this study. Awareness on ALS among some practicing medical officers could not be assessed as they did not come forward to respond to this questionnaire. Though data collection was done by a self-administered standardized validated questionnaire (Appendix 1) prepared based on the United Kingdom Resuscitation Council Guidelines on ALS. This is the first study for which a questionnaire was used and since this is a single centre study, it is not advisable to be generalized to other settings.

## Conclusions

Overall, just over 10 % of participants demonstrated inadequate ALS knowledge scores. A significantly higher proportion of final year medical students had good knowledge, compared to medical officers and 4th year students. Only one-third of participants were confident in saving a life with their current ALS knowledge. Nearly all participants thought that the ALS course should be reevaluated frequently. ALS teaching and training which starts at the undergraduate period, should be frequently and regularly revised, with continuous medical education (CME), along with the testing of knowledge and skills at regular intervals. Thus, in order to improve knowledge, skills and confidence among doctors, and to keep them up to date with any revision of the guidelines. Also large multicentre studies are needed to confirm the results of this type of preliminary study.

## Abbreviations

ALS: advanced life support
CPR: cardio-pulmonary resuscitation
BLS: basic life support

## References

[1] Chandrasekaran S, Kumar S, Bhat SA (2010). Awareness of basic life support among medical, dental, nursing students and doctors. Indian J Anaesth.

[2] Zaheer H, Haque Z (2009). Awareness about BLS (CPR) among medical students-status and requirements. J Pak Med Assoc.

[3] Narayan DPR, Biradar SV, Reddy MT, Bk S (2015). Assessment of knowledge and attitude about basic life support among dental interns and postgraduate students in Bangalore city, India. World J Emerg Med.

[4] Sharma R, Attar NR (2012). Adult basic life support (BLS) awareness and knowledge among medical and dental interns completing internship from deemed university. NUJHS.

[5] Deakin CD, Nolan JP, Soar J (2010). European resuscitation council guidelines for resuscitation 2010. Section 4. Adult advanced life support. Resuscitation.

[6] Mackay J, Mensah GA, Mendis S, Greenlund K, World Health Organization (2004). The atlas of heart disease and stroke.

[7] Field JM, Hazinski MF, Sayre MR, Chameides L, Schexnayder SM, Hemphill R (2010). Part 1: executive summary: 2010 American Heart Association guidelines for cardiopulmonary resuscitation and emergency cardiovascular care. Circulation.

[8] Businger A, Rinderknecbt S, Blank R, Merki L, Carrel T (2010). Students’ knowledge of symptoms and risk factors of potential life-threatening medical conditions. Swiss Med Wkly.

[9] Bhanji F, Mancini ME, Sinz E (2010). Part 16: education, implementation, and teams: 2010 American Heart Association Guidelines for cardiopulmonary resuscitation and emergency cardiovascular care. Circulation.

[10] Soar J, Monsieurs KG, Balance JHW (2010). European Resuscitation Council guidelines for resuscitation 2010. Section 9. Principles of education in resuscitation. Resuscitation.

[11] Soar J, Mancini ME, Bhanji F (2010). Part 12: education, implementation, and teams: 2010 International consensus on cardiopulmonary resuscitation and emergency cardiovascular care science with treatment recommendations. Resuscitation.

[12] Avabratha KS, Bhagyalakshmi K, Puranik G, Shenoy KV, Rai BS (2012). A study of knowledge of resuscitation among interns. Al Ameen J Med Sci.

[13] Almesned A, Almeman A, Alakhtar AM (2014). Basic life support knowledge of healthcare students and professionals in the Qassim University. Int J Health Sci.

[14] Akritia S, Mayankb M, Arushic D (2014). Basic life support and advanced cardiac life support: knowledge of medical students in New Delhi. J Young Med Res.

[15] Chandraguptha S (2011). An audit on knowledge of intern house officers on cardio pulmonary resuscitation. Sri Lankan J Anaesthesiol.

[16] Hammond F, Saba M, Simes T (2000). Advanced life support: retention of registered nurses’ knowledge 18 months after initial training. Aust Crit Care.

[17] Birnbaum ML, Robinson NE (1994). Effect of advanced cardiac life support training in rural, community hospitals. Crit Care Med.

[18] Yang CW, Yen ZS, McGowan JE, Chen HC, Chiang WC, Mancini ME, Soar J, Lai MS, Ma MH (2012). A systematic review of retention of adult advanced life support knowledge and skills in healthcare providers. Resuscitation.

[19] Roshana S, Batajoo KH, Piryani RM, Sharma MW (2012). Basic life support: knowledge and attitude of medical/paramedical professionals. World J Emerg Med.

[20] Sudeep CB, Sequeira PS, Jain J, Jain V, Maliyil M (2013). Awareness of basic life support among students and teaching faculty in a dental college in Coorg, Karnataka. Int Dent J Stud Res.

[21] Jensen ML, Lippert F, Hesselfeldt F (2009). The significance of clinical experience on learning outcome from resuscitation training—a randomised controlled study. Resuscitation.

[22] Vidal SA, Ronfani L, da Mota Silveira S (2001). Comparison of two training strategies for essential newborn care in Brazil. Bull World Health Organ.

[23] Kromann CB, Bohnstedt C, Jensen ML, Ringsted C (2009). The testing effect on skills learning might last 6 months. Adv Health Sci Educ Theory Pract.

[24] Kromann CB, Jensen ML, Ringsted C (2009). The effect of testing on skills learning. Med Educ.

[25] Rajapakse BN, Neeman T, Dawson AH (2013). The effectiveness of a ‘train the trainer’ model of resuscitation education for rural peripheral hospital doctors in Sri Lanka. PLoS One.

